# Assessing safety trends of withdrawn medications: A data-driven pharmacovigilance approach using growth models

**DOI:** 10.1016/j.rcsop.2026.100719

**Published:** 2026-02-14

**Authors:** Samadhan Ghubade, Sharvari Shukla

**Affiliations:** Symbiosis Statistical Institute, Symbiosis International (Deemed University), Pune, India

**Keywords:** Adverse drug reactions (ADRs), Model fitting, Withdrawn drugs, Pharmacovigilance, World Health Organization (WHO), Detriment index, Growth rate

## Abstract

**Background:**

Adverse drug reactions (ADRs) are critical in evaluating a medicine's safety profile during development and post-marketing surveillance. This study focuses on medications linked to major adverse drug reactions (ADRs) that were later taken off the market or removed from usage. Adverse drug reaction (ADR) reports were gathered from VigiAccess and the U.S. FDA (United States Food and Drug Administration) Adverse Event Reporting System (FAERS). Data visualization tools were then used to evaluate how reporting patterns changed over time. Although a decrease in adverse drug reaction (ADR) reports after a withdrawal may be expected, we observed various patterns over time. These included sigmoidal, exponential, and linear trends, which indicated that safety signals persisted differently. Assessment was performed using cumulative annual ADR reporting trends, supplemented by published evidence on typical latency intervals between drug exposure and event onset for the medicines of interest. Additionally, we developed a novel statistical metric termed the Detriment Index, based on curve-fitting and growth-rate modeling, to quantitatively compare the relative safety of drugs. This approach allows ranking of medicines with respect to ADR accumulation, supports identification of safer therapeutic alternatives, and provides practical decision support for clinicians and regulators.

A total of 39 withdrawn medications were included based on regulatory withdrawal records documented by WHO and FDA sources. For comparative safety assessment, 15 commonly used cancer medications (including Tamoxifen, Avastin, Bleomycin, Paclitaxel, Vincristine, Methotrexate, Cisplatin, Doxorubicin, Imatinib, Docetaxel, Rituximab, Trastuzumab, Revlimid, Lenalidomide, and Pembrolizumab) were analyzed. Four distinct growth patterns were identified based on model fit: Saturation (*n* = 17), Linear (*n* = 8), Exponential (*n* = 9), and Sigmoidal (*n* = 5), with overall model performance demonstrating strong goodness-of-fit (R^2^ = 0.83–0.97). To enable comparative interpretation of safety profiles, we introduce composite, model-based metric derived from the fitted cumulative ADR curve called ‘Detriment Index,’ which integrates the ADR growth-rate parameter (β) with the cumulative burden (area-under-curve) of ADR reports, providing a normalized metric that captures both the speed and magnitude of safety-signal accumulation. This allows comparative safety ranking across drugs regardless of differences in reporting patterns or curve shapes. Detriment Index integrates both the speed of ADR accumulation and the total accumulated harm, enabling comparative safety assessment across drugs.

To understand the context of these withdrew drug trends compared to currently used pharmaceuticals, we investigated group of active oncology drugs. In contrast, the occurrence of adverse drug reactions (ADRs) with tamoxifen increased more slowly, but pembrolizumab showed a quicker and more consistent rise in these events. These differences show that combining trend categorization with the Detriment Index improves our understanding of safety and boosts post-marketing surveillance systems. The findings show that reporting adverse drug reactions (ADRs) continues, even after a medicine is removed from the market, also show how using quantitative methods can improve safety assessments and support clinical decisions.

**Methods:**

For analysis publicly available datasets from VigiAccess, the WHO's global database of reported potential side effects of medicinal products, and the FDA's Adverse Event Reporting System (FAERS) Public Dashboard have been used. A comprehensive analysis was performed on cumulative count of ADR reports for selected medications that have been withdrawn from the market. The investigation focused on finding patterns in the number of adverse drug reaction (ADR) reports related to the termination of these drugs. To visualize these trends, different curve-fitting methods have been used. These included both linear and non-linear statistical models, such as sigmoidal, exponential, and linear forms. A ranking was also made using an exponential growth rate model to compare the safety ratings of different cancer drugs. This model was used to look at and compare the drug's safety features.

**Results:**

When a medication is withdrawn or banned, its utilization is anticipated to cease, resulting in the absence of further reports of adverse drug reactions (ADRs). This is the predicted result for all pharmaceuticals that have been banned. The analysis of 39 medications indicated diverse linear and nonlinear patterns. Specifically, 17 drugs followed a saturation pattern, 10 showed a linear pattern, 7 exhibited an exponential pattern, and 5 displayed a sigmoidal pattern. The examples presented in this investigation demonstrated that the drugs Benoxaprofen, Rosiglitazone, Temazepam, and Rofecoxib exhibited a robust correspondence with diverse modeling approaches. Specifically, Benoxaprofen conformed to a saturating hyperbola model, achieving a R^2^ value of 0.98. Rosiglitazone, conversely, was best represented by a linear model, which yielded a R^2^ value of 0.96. Furthermore, Temazepam displayed an exceptional fit with the exponential model, resulting in a R^2^ value of 0.99. The data for rofecoxib showed a sigmoidal pattern, with a R^2^ value of 0.92, indicating a strong fit to the sigmoidal model. In a safety comparison of fifteen oncological agents, Tamoxifen demonstrates a more favorable safety profile, attributable to its reduced rate of ADR accumulation and a lower growth rate of 0.0972. Conversely, Pembrolizumab exhibits a higher exponential growth rate of 0.8277, which suggests an increased risk profile.

**Conclusions:**

This study shows that adverse drug reaction (ADR) patterns, particularly those for banded/withdrawn drugs and some cancer therapies, demonstrate how safety issues alter over time. Adverse drug response (ADR) reports normally diminish after a drug is discontinued, however the rate varies. This pattern shows historical risk estimates, not those medications are safer.

Analyzing the overall patterns in adverse drug reactions (ADRs) provides an additional viewpoint to established ways of monitoring drug safety. This approach allows for a more nuanced understanding of long-term safety data. The Detriment Index, a new metric based on trends, allows for the comparison of the long-term burden of adverse drug reactions (ADRs) across different medications, regardless of their regulatory history or how they are used in treatment. This method helped separate medications with lower risks, like Tamoxifen, from others that build up in the body more quickly, such as Pembrolizumab. This shows its potential for comparing safety profiles.

This exploratory approach, in essence, enriches our understanding of how long post-marketing safety information lasts. It also provides a foundation for future research. This research could help support safer clinical decisions and regulatory evaluations for both pharmaceuticals that are no longer on the market and those that are still available.

## 1 Introduction

All allopathic medications have the potential to have negative side effects.^1^ Prior to authorizing a medication, pharma regulators assess its advantages against any potential negative side effects.^2^ While evaluating the safety and effectiveness of the medications, adverse drug reactions (ADRs) are a major concern in the medication development process.^3^ Data from the drug's clinical trials (CTs) is used to support its approval. But after the medication is licensed and sold, side effects that weren't seen in the initial studies may surface. This is due to the fact that CTs have time, subject count, ethnicity, age, health composition, and other restrictions.^4^ It is expected of pharmaceutical corporations to continuously track the side effects that their products cause to patients.^5^

Adverse drug reactions (ADRs) are unpleasant occurrences that may have resulted from a medication or prescription. In the real world, these ADRs are notified to the holders of market authorizations (MAH) and health authorities (HA) concurrently. The MAH and HAs then archive these ADRs. The WHO's “Vigibase” and the US FDA's adverse event reporting system (FAERS) are the two main databases containing these reports. Millions of these ADRs are contained in these databases.

One sort of surveillance known as pharmacovigilance mostly depends on voluntary ADR reporting. One well-known public source for such data is The Uppsala Monitoring Centre of the World Health Organization (WHO), accessible through their website www.vigiaccess.org, which offers annual ADR report counts for various medications. Additionally, the FDA provides an overview of ADRs through their Questions and Answers on the FDA's Adverse Event Reporting System (FAERS).

As anticipated, ADR data undergo thorough and detailed analysis to identify patterns, assess drug safety, and understand the potential risks associated with medications. Such analyses contribute to regulatory decision-making, safety communication, and public health.^6^, ^7^, ^8^, ^9^, ^10^ Previous studies across regions (e.g., SSA, India) highlight persistent underreporting, heterogeneity, and limitations in pharmacovigilance systems.^7^, ^8^, ^9^, ^10^

A few research projects used the Weber model, which suggested that spontaneous reports peak in the first two years after market introduction and decline thereafter. Safety signal detection is another major goal, and disproportionality analysis is commonly used.^11^, ^12^, ^13^, ^14^ Analysis carried out for the complexity and risks of drug-drug interactions in oral chemotherapeutic cancer treatments by studying related clinical outcomes.^15^ These approaches typically evaluate one drug–ADR pair at a time, limiting broader understanding of long-term safety trajectories.^16^, ^17^

However, a crucial gap remains insufficiently explored: how ADR reporting patterns evolve over time, including after a drug is withdrawn from the market. Post-withdrawal reporting may continue due to latent ADR manifestation, delayed reporting behavior, or continued off-label/stock-based use. Understanding whether ADR accumulation attenuates, stabilizes, or persists long after withdrawal is essential for evaluating real-world safety risk, communicating regulatory decisions, and improving pharmacovigilance responsiveness. This study specifically addresses this gap by examining long-term ADR trend patterns for both active and withdrawn products to determine how persistent safety signals behave over time.

To guide this investigation, the study adopts the following conceptual pathway: ADR data → temporal trend patterns → safety signal persistence → Detriment Index → comparative safety assessment. This framework enables a macro-level evaluation of safety evolution across drugs, supporting clearer interpretation of long-term risk behavior beyond individual signal detection metrics.

Post-marketing signal detection traditionally relies on disproportionality methods such as the Proportional Reporting Ratio (PRR), where thresholds like PRR ≥ 2 and χ^2^ ≥ 4 are used to flag potential signals.^18^ The WHO's VigiBase uses the Bayesian Confidence Propagation Neural Network (BCPNN) to compute the Information Component (IC), where IC₀₂₅ > 0 indicates a statistically supported signal.^19^ Although valuable for signaling specific drug–event combinations, these approaches do not evaluate temporal persistence of ADR accumulation. The present study complements this established literature by shifting focus from disproportionality to trend-based analysis, emphasizing cumulative reporting behavior over time particularly for withdrawn drugs to understand broader patterns of safety evolution.

By integrating trend analysis, persistence evaluation, and the proposed Detriment Index (a summary indicator of ADR growth behavior across time), this study aims to provide a clearer comparative safety perspective across oncology drugs, including those withdrawn for safety reasons. This aligns directly with the study's objective of offering new perspectives on the safe use of medications through long-term post-marketing behavior rather than isolated signal detection.

### 1.1 Study objectives and research questions

This study aims to fill the current knowledge gaps in how adverse drug reaction (ADR) reporting works and main study objectives are as:

• To characterize temporal patterns in cumulative adverse drug reaction (ADR) reporting before and after market withdrawal using publicly available pharmacovigilance data (e.g., VigiAccess, FAERS).
• To develop and define the Detriment Index as an exploratory metric that summarizes the persistence, or escalation of ADR reporting over time, particularly after product withdrawal.
• To apply the Detriment Index for comparative safety assessment across selected drugs, including both withdrawn medicines and active oncology products, to illustrate how long-term ADR trends differ beyond traditional disproportionality signal detection methods.

## 2 Methods

### 2.1 Data collection, processing and analysis

Two categories of medicines were included in the analysis:

1. **Withdrawn / Banned Drugs (*n* = 39):**
  These medicines were removed from the market due to safety concerns. The withdrawal list was compiled using WHO pharmacovigilance withdrawal documents and U.S. FDA market withdrawal records.
2. **Active Oncology Drugs (*n* = 15):**
  Commonly prescribed cancer therapies were selected based on clinical use, global prescribing prevalence, and the availability of ADR data from VigiAccess and FAERS. These drugs were included to demonstrate the comparative safety assessment capability of the Detriment Index.

### 2.2 Rationale for mixing withdrawn and active drug datasets

This study intentionally integrates data from both **withdrawn** and **active oncology** drugs to create a diverse safety spectrum for model evaluation.

• Withdrawn drugs typically show rapid ADR buildup and strong historical safety signals**.**
• Active oncology drugs show ongoing and evolving ADR reporting patterns, reflecting real-world pharmacovigilance complexity.

Including both categories enables:

• Stronger contrast between low and high-risk trajectories,
• Improved robustness of the Detriment Index across different lifecycle stages, and
• Greater external validity, as regulators routinely compare signals across drugs that are active, restricted, or discontinued.

This mixed dataset therefore better reflects real-world regulatory decision-making**,** where safety assessments occur across heterogeneous product histories.

### 2.3 Classifying regulatory withdrawals

To increase interpretability each banned medicine was categorized. Definitions of three mutually exclusive categories: Withdrawn in most or all main regulatory regions, regionally banned—only in specific countries or regions, some formulations, dosages, were removed, but others remained.

This classification ensured analytical consistency and sophisticated post-withdrawal reporting pattern comparison. This study will fill gaps in knowledge on adverse drug reaction (ADR) reporting after a drug is withdrawn.

### 2.4 Database

Adverse drug reaction (ADR) reports were obtained from two publicly available pharmacovigilance databases:

1. **VigiAccess (maintained by WHO-Uppsala Monitoring Centre)** and
2. **FAERS (maintained by U.S. Food and Drug Administration)**.

For each drug, ADR counts were extracted annually from the year of initial marketing approval up to 2023, including the time period after market withdrawal, where applicable.

### 2.5 Data cleaning and standardization

To maintain reproducibility and consistency, data-processing steps were applied uniformly across all drugs. Duplicate records were removed, and non-drug entries were excluded. For FAERS, multiple files corresponding to the same calendar year were merged to produce a single annual count.

### 2.6 Cumulative ADR construction

Instead of evaluating isolated yearly ADR counts, cumulative ADR curves were generated for each drug. This method:

1. Smooths natural variability in annual reporting and highlights long-term growth patterns.
2. Aligns with the study's goal of understanding overall reporting trajectories rather than year-to-year fluctuations.
3. Allows clear comparison of post-withdrawal persistence, minimizing the influence of short-term stimulated reporting spikes.

For each drug, the x-axis represented time in years, and the y-axis represented cumulative ADR reports aggregated annually.

Although the expected trend for a withdrawn drug is a saturation (plateau) curve, several medications displayed linear, exponential, or sigmoidal shapes. These deviations were of primary analytical interest.

### 2.7 Reproducibility and transparency

To enhance reproducibility, the analysis workflow included:

**Figure:**
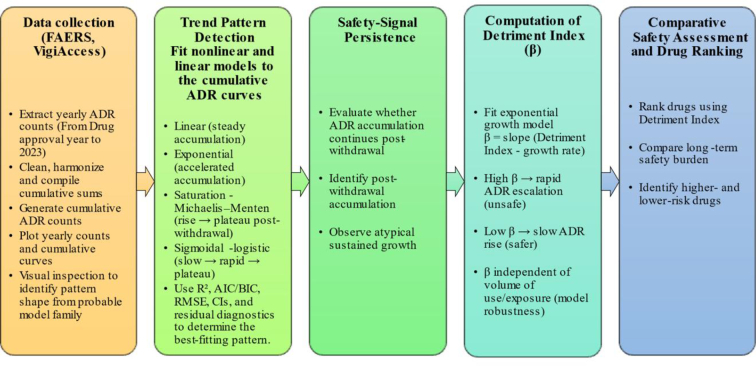

### 2.8 Equation (exponential growth model)

To quantify the long-term accumulation pattern of adverse drug reaction (ADR) reports, we used the exponential growth model:

$$Y\left(t\right)=\alpha *\exp\left(\beta^{t}\right)$$

Where,

• *Y(t)* = cumulative ADR count at time *t*,
• *α* = scaling parameter,
• *β* = growth-rate parameter,
• *β* is defined as the Detriment Index.

While the Detriment Index is mathematically equal to β, it is conceptually distinct since β is reinterpreted, reframed, and validated as a comparative medication risk assessment with specific attributes:

1. β must reflect the intrinsic accumulation tendency of ADRs**,** not reporting volume or market size.
2. β must remain stable under proportional changes in drug usage or exposure.
3. β must allow ranking drugs on a consistent scale**,** irrespective of therapeutic area, market size, or population exposure.

Apart from Linear, Quadratic, polynomial or any other nonlinear model the exponential model is required since it has key property of independence from drug usage or exposure volume:

$$2Y\left(t\right)=2\alpha *\exp\left(\beta^{t}\right)$$

Multiplying all observations by any constant *k* affects *α* but does not change β.

Thus:

• β remains stable even if a drug is widely used or rarely used.
• β captures growth behavior, not scale.
• Therefore, β is a valid safety-comparison index.

**Table:** 

Model	Issue
Linear (Y = a + bt)	When ADR counts double, slope b doubles. Depends on usage volume unacceptable for cross-drug comparison
Quadratic / Cubic	The parameters scale with total exposure and cannot be ranked for risk.
Generic nonlinear models	Parameters are not invariant to multiplicative scaling

Thus, only the exponential model provides a scale-invariant growth parameter suitable for ranking**.**

**Interpretation of the Detriment Index**

• Higher β **→** faster “explosion” of ADR counts **→** more hazardous drug
• Lower β **→** slow accumulation **→** safer profile
• β = 0 **→** completely flat cumulative curve **→** no new ADRs**,** ideal safety behavior

This yields a straightforward, clinically interpretable severity metric.

The novelty of the Detriment Index does not come from the mathematics of β itself, but from redefining β as a standardized, interpretable cross-drug safety metric. This study uniquely applies β to quantify safety signal persistence across drugs with very different reporting volumes, therapeutic classes, and withdrawal patterns.

By using β as a Detriment Index, the approach:

• Enables consistent comparison between withdrawn and active drugs regardless of scale or stimulated reporting.
• Identifies drugs with persistent long-term safety concerns, supporting regulatory prioritization.
• Functions as a decision-support tool for ranking drugs based on cumulative harm patterns.

Thus, while β is mathematically simple, its new interpretation, validation, and application for post-marketing safety assessment represent the true innovation of this work.

### 2.9 Model fitting and computation – Software and reproducibility

• **R (v4.3) & Python (v3.9)** – Used for curve fitting, model estimates, AIC/BIC computation, residual diagnostics plots, and reproducible analytical workflows.
• **Minitab 17** – Used for curve-fitting plots.
• **SAS 9.4** – Used for dataset cleaning, data organization, and nonlinear model fitting.

**Table on Dataset Description, Source, Modeling Approach, and Fit Diagnostics for Four Representative Drugs described in** Section 2.9.1 **to** Section 2.9.4**.**

**Table:** 

Dataset	Source	Period Extracted	Drugs (n)	Pattern Criteria	Model Applied	Fit Statistics Reported	Remarks
Drug A (Saturation pattern) Benoxaprofen	FAERS	1982–1999 (Yearly counts extracted)	1	Rapid increase followed by plateau in cumulative ADR; visually resembles enzyme-kinetic saturation behavior	Michaelis–Menten (Nonlinear saturation model)	RMSE, AIC/BIC, R^2^, parameter CI, model diagnostics plots	Approaches asymptote; suitable for drugs with exhausted reporting momentum
Drug B (Linear pattern) Rosiglitazone	VigiAccess	2004–2022	1	Approximately constant year-on-year increase in ADR reporting; no curvature	Linear regression	Slope ± SE, RMSE, R^2^, AIC/BIC, residual diagnostics	Useful for drugs with stable reporting; minimal acceleration
Drug C (Exponential pattern) Temazepam	VigiAccess	1980–2023	1	Yearly ADR counts show multiplicative growth; cumulative ADR curve rises sharply	Exponential model	Growth rate β ± CI, RMSE, AIC/BIC, R^2^, residual plots	β interpreted as Detriment Index; sensitive to persistent safety signals
Drug D (Sigmoidal pattern) – *Rofecoxib*	FAERS	1999–2023	1	Slow initial ADR rise → rapid escalation → plateau; classic S-shaped trajectory	Sigmoidal / Logistic growth model	RMSE, AIC/BIC, R^2^, CI for upper asymptote and inflection	Reflects lifecycle-driven reporting surges; ideal for withdrawn drugs

#### 2.9.1 Saturating growth

When a drug is banned, its usage is expected to cease, leading to a drop in adverse drug reactions (ADRs) to zero. This cessation of ADRs should result in a flat cumulative count over time, which is the anticipated pattern for all banned drugs, indicating that no new ADR reports are being generated post-ban.

An example of a drug following this saturating curve is Benoxaprofen. Developed and marketed by Eli Lilly and Company, an American pharmaceutical firm, Benoxaprofen was used for its anti-inflammatory and analgesic properties to treat conditions like rheumatoid arthritis and osteoarthritis. The U.S. Food and Drug Administration (FDA) approved it in 1982, but it was withdrawn from the market within the same year due to serious side effects, including hepatotoxicity (liver damage), photosensitivity reactions, and renal impairment.

**Fig. 1:**
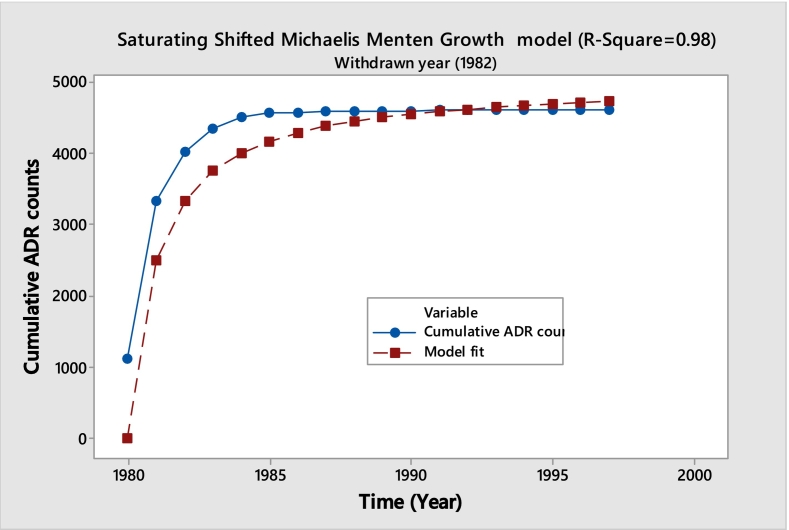
Benoxaprofen (Cumulative ADR Observed vs. Fitted).

The below given **(**Fig. 1**)** displays the observed accumulation curve and an R^2^ value of 0.98 indicates a decent fit with a saturating hyperbola fitted to the data. The saturating fit, which shows that the frequency of ADR reports has sharply dropped and to insignificant levels following the withdrawal, provides insight into the withdrawal's effectiveness. This identical saturation trend is present in about seventeen different medications. An inventory of these medications is included in the Table 1. This pattern shows a consistent drop in ADR complaints after the ban, demonstrating the drug's successful withdrawal from use.

**Table 1:** Drugs prohibited based on ADR accumulation count curve form.

Saturation	Linear	Exponential	Sigmoidal
Belviq	Dofetilide	Bromhexine	Rofecoxib
Natalizumab	Flunitrazepam	Hydromorphone	Aprotinin
Pergolide	Ketorolac	Ibuprofen	Lumiracoxib
Phenylbutazone	Ozogamicin	Iproniazid	Redux
Tegaserod	Rosiglitazone	Amphetamine	Roxiam
Terfenadine	Clobutinol	Metamizole	
Troglitazone	Destibenol	Temazepam	
Benoxaprofen	Cervidil		
Bromfenac	Tegison		
Simvastatin	Phenylbutazone		
Cisapride			
Eculizumab			
Nefazodone			
Meridia			
Trovafloxacin			
Efalizumab			
Valdecoxib			

Saturating Growth Shifted Michaelis Menten model

$$Y=Y_{0}+\frac{{ϴ}_{1}*X}{{ϴ}_{2}+X}+\varepsilon$$

Saturating growth shifting Michaelis–Menten model is selected since data start with a non-zero baseline and classical model pressures cause systematic residual patterns and biased parameter estimates. This misspecification is fixed by adding a baseline term Y_0_, improving fit quality, residual diagnostics, and inference.

**Table:** 

Shifted Michaelis Menten Parameter Estimates for Benoxaprofen				
Parameter	Estimate	Standard Error	Lower 95% CI	Upper 95% CI
Y0	1106.6828	85.4586	939.1839	1274.1817
ϴ1	3694.5278	90.5612	3517.0278	3872.0279
ϴ2	0.541521	0.0543	0.4350	0.6479

Model Fit Statistics for Benoxaprofen	
Metric	Value
AIC	162.8885
BIC	165.5596
RMSE	78.1019
R-squared	0.98

CI=Confidence Interval.

**Model Diagnostics plots for Benoxaprofen**

**Figure:**
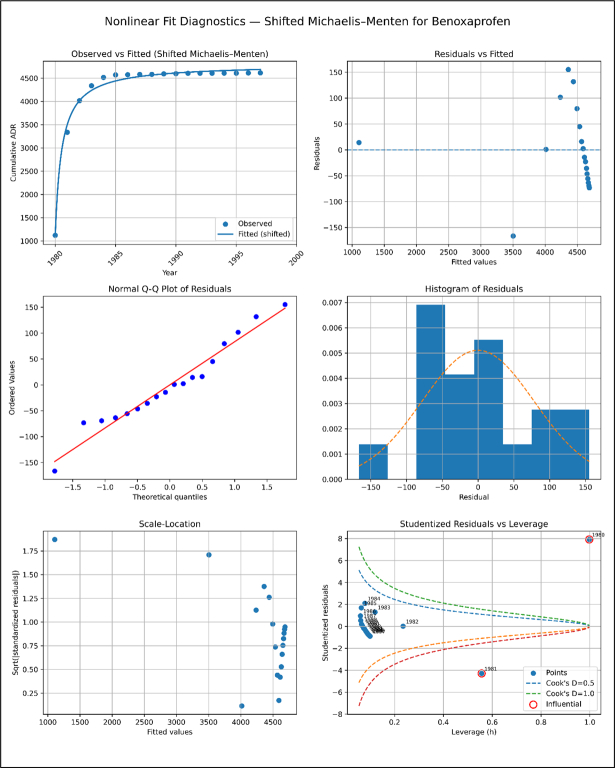

The shifted Michaelis–Menten model has better fit quality, residuals around zero, reduced curvature, and a Q–Q plot that matches the theoretical line. Except for a few high-leverage spots, fitted data show more uniform variance and no serious model assumptions violations. Diagnostics show that the shifted model presents cumulative ADR data reasonably and reliably.

#### 2.9.2 Linear growth

In the analysis, we identify ten drugs exhibiting a linear pattern shown in Table 1. As discussed earlier, the primary aim is to identify drugs that deviate from the expected saturating pattern. A notable outlier is Rosiglitazone, developed and marketed by GlaxoSmithKline (GSK) for the treatment of type 2 diabetes mellitus. This drug was approved by the U.S. Food and Drug Administration (FDA) in 1999. However, in 2010, the European Medicines Agency (EMA) suspended its marketing authorization due to concerns regarding cardiovascular safety. Although these restrictions were lifted in 2013 following a re-evaluation of safety data, the usage of Rosiglitazone had already declined significantly by then.

**Fig. 2:**
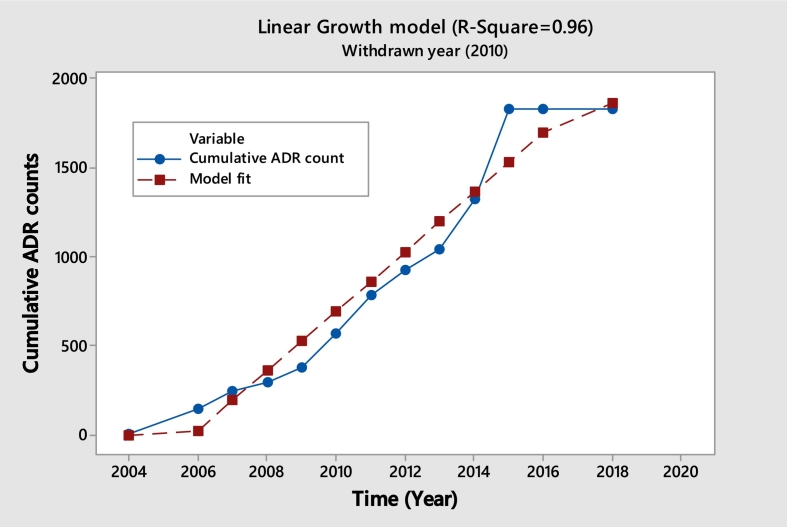
Rosiglitazone (Cumulative ADR Observed vs. Fitted).

A well-fitting linear model with an R^2^ value of 0.96 is shown in Fig. 2. The yearly count of adverse drug reaction (ADR) reports appears to have steadied at about 155, according to this linear fit. A continuous volume of intake and a constant probability of adverse responses per usage are suggested by the consistent annual ADR report count. This steady rate of ADR reports aligns with the idea that while the restrictions on Rosiglitazone were lifted, its withdrawal might have been only partial, allowing its continued use in some regions. Thus, drugs like Rosiglitazone, which exhibit this linear reporting pattern, highlight a steady incidence of ADRs despite regulatory changes.

**Table:** 

Linear Model Parameter Estimates for Rosiglitazone					
	Coefficients	Standard Error	t Stat	Lower 95% CI	Upper 95% CI
Intercept	2005.7857	0.4922	4074.925	2004.7023	2006.8690
Slope	0.0060	0.0004	13.2036	0.0050	0.0070

Model Fit Statistics for Rosiglitazone	
Metric	Value
AIC	172.2
BIC	173.3
RMSE	156.0762
R-squared	0.96

CI=Confidence Interval.

**Model Diagnostics plots for Rosiglitazone.**

The diagnostic plots indicate that the linear model fits this dataset very well, with residuals showing no major violations of linear regression assumptions and a very high R^2^ value confirming an excellent linear relationship. Overall, the model adequately captures the trend in cumulative ADR growth across the years.

.

#### 2.9.3 Exponential growth

**Fig. 3:**
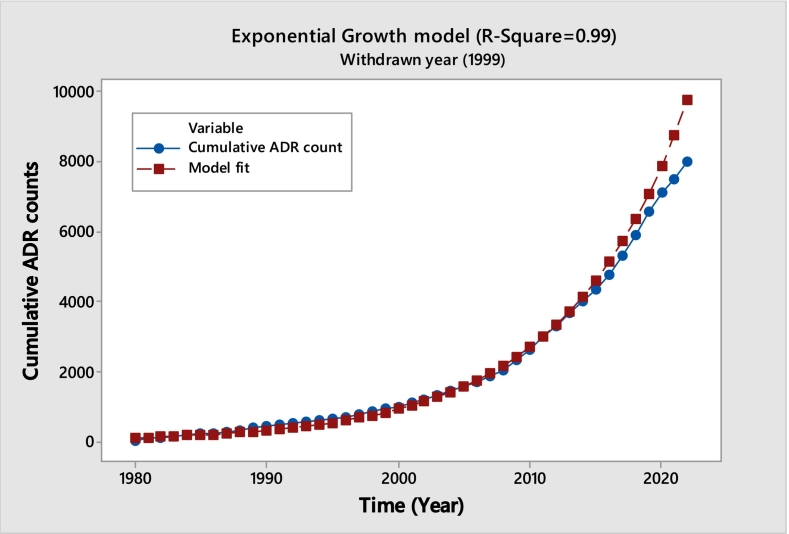
Temazepam (Cumulative ADR Observed vs. Fitted).

In our next case study, we examine the drug **Temazepam**, which was approved by the U.S. Food and Drug Administration (FDA) in 1981. Initially marketed by Hoffmann-La Roche under the brand name Restoril, it was removed from the market in 1999 due to issues related to diversion, abuse, and a higher incidence of overdose deaths compared to other drugs in its class. The data presented in Fig. 3 provides an exponential model with an R^2^ value of 0.99, indicating a strong fit. This model reveals a dramatic increase in the number of adverse drug reaction (ADR) reports, surpassing 2000 by 2023. This data suggests that the previous explanations do not apply in this case. Although temazepam remains available and is prescribed for short-term use due to its potential for dependence and other side effects, its consumption has increased systematically. Temazepam has also been linked to incidences of date rape and reported to be addictive.

**Table:** 

Exponential Model Parameter Estimates for Temazepam			
	Estimate	Standard Error	95% CI
Intercept	108.5076	4.6868	(88.7872, 132.6081)
growth rate	0.107054	0.1071	(0.098832, 0.115277)

Model Fit Statistics for Temazepam	
Metric	Value
AIC	28.9828
BIC	32.5052
RMSE	381.0800
R-squared	0.99

CI=Confidence Interval

**Model Diagnostics plots for Temazepam.**

**Figure:**
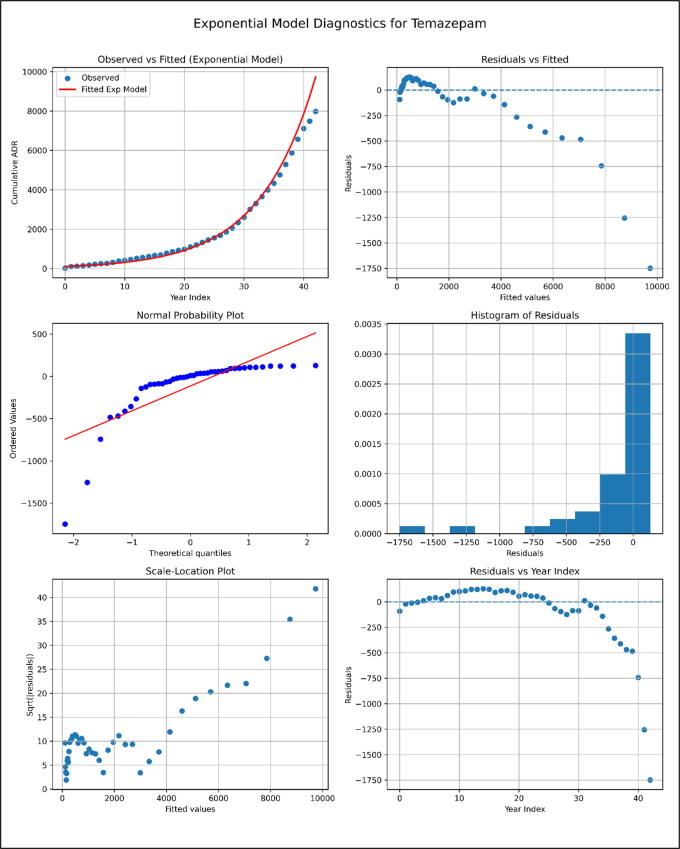

The exponential growth model provides an excellent fit for the Temazepam cumulative ADR data, with strong goodness-of-fit metrics and diagnostics confirming an appropriate exponential pattern in the residual structure.

#### 2.9.4 Sigmoidal growth

**Fig. 4:**
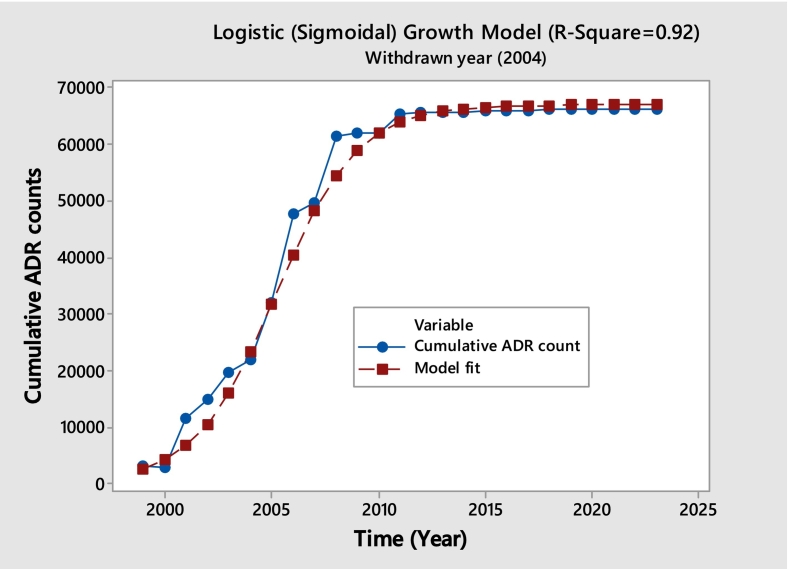
Rofecoxib (Cumulative ADR Observed vs. Fitted).

In our analysis of the drug Rofecoxib, a nonsteroidal anti-inflammatory drug (NSAID) developed by Merck & Co., which was approved by the FDA in 1999 and withdrawn from the market in 2004 due to cardiovascular risks, we observed a notable trend. Following its withdrawal, before showing indications of leveling off, the number of adverse drug reaction (ADR) reports increased for almost eight years. This trend is represented by a sigmoidal pattern, as demonstrated by an R^2^ value of 0.92 and an excellent fit displayed in Fig. 4. The continued increase in ADR reports post-withdrawal is unexpected. We have identified six other drugs exhibiting a similar sigmoidal pattern (refer to the Table 1), highlighting that while this pattern resembles a saturating trend, there are distinct characteristics to be noted. Saturation is evident in this category as well, although it manifests differently compared to other patterns.

**Table:** 

Logistic (Sigmoidal) Growth Model Parameter Estimates for Rofecoxib				
Parameter	Estimate	Standard Error	Lower 95% CI	Upper 95% CI
k (growth rate)	0.5513	0.0318	0.4853103	0.617323
t0 (midpoint)	5.7469	0.1192	5.499718	5.994114

Model Fit Statistics for Rofecoxib	
Metric	Value
AIC	386.9552
BIC	390.6119
RMSE	2036.7383
R-squared	0.92

CI=Confidence Interval.

**Model Diagnostics plots for Rofecoxib.**

**Figure:**
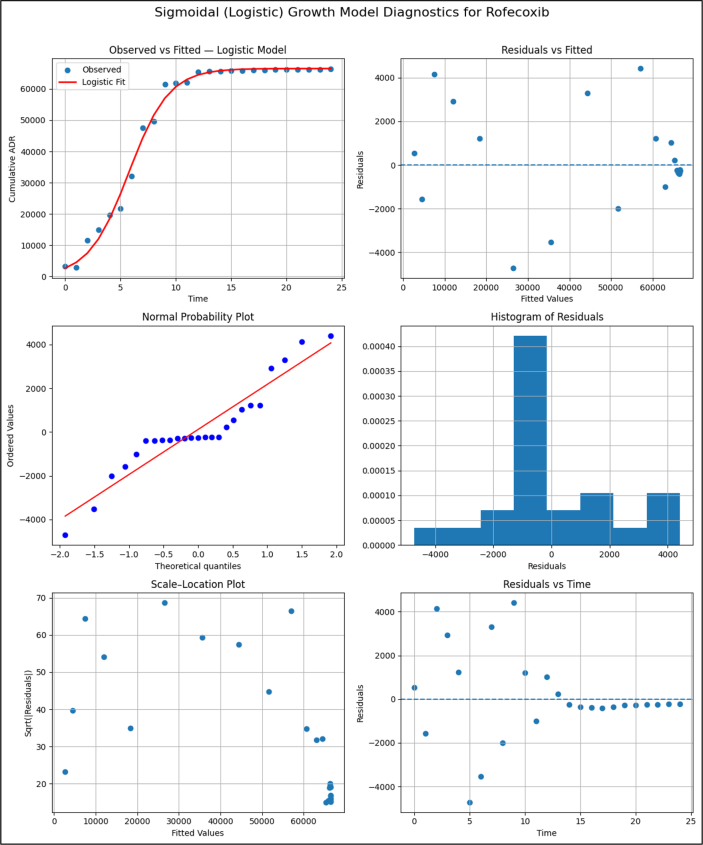

The diagnostic plots indicate that the logistic model provides a good overall fit to the sigmoidal ADR trajectory, adequately capturing the rapid growth and subsequent plateau despite moderate residual variability.

Although detailed plots and parameter estimates are shown only for representative examples of each curve type, based on the model-fitting analysis carried out for each drug, the following classification summarizes which medications shown saturation, linear, exponential, or sigmoidal ADR accumulation patterns. (Table 1).

## 3 Method of curve fitting and medication safety comparison

The ADR numbers for pharmaceuticals that are reported for drugs that are discontinued or prohibited are expected to follow the saturation model when totaled. Surprisingly, yet, some of these medications rise according to linear, exponential, or sinusoidal patterns. This might be the result of bulk reporting of ADRs that were previously unreported or of reporting incentives.

Sometimes, people may continue taking a medication despite negative reports if they have a positive.

It is possible to use the current curve fitting technique to currently prescribed medications and possibility of modeling using comparatively simple methods. Working with de-identified data and examining trends for important organ classes including the heart, lungs, liver, and kidneys will also be pertinent.^18^ If necessary, the list of adverse events can be reduced to only those that are crucial or clinically meaningful. The primary worry is whether the assortment of forms included in the category of medications that are restricted will be sufficient to encompass the larger group.

Study to identify the patterns and describe the uses of artificial intelligence in pharmacovigilance through a systematic literature review and hence comparing drug safety profiles is a useful use of pattern recognition in adverse drug reactions (ADRs) in pharmacovigilance research.^20^ When evaluating drugs with comparable efficacy—i.e., those offering similar therapeutic benefits for the targeted condition—preferentially selecting the drug with a more favorable ADR profile becomes crucial.

A single drug may be associated with a wide array of adverse reactions, comparisons must rely on summary measures to distill complex ADR data.^21^, ^22^ For example, when a drug shows a linear increase in ADR report counts over time, the slope of this line serves as a summary statistic. The average annual increase of ADR reports is seen by this slope. In theory, a steeper slope would indicate a better safety profile for medications with comparable efficacy and linear growth in ADR counts. However, interpreting this measure requires caution due to variations in drug exposure, which can significantly influence ADR counts. A drug with higher exposure will naturally accumulate more ADR reports over time.

Think about two medications, Test drug and Reference drug, for example. If Reference drug sells 50 ADR reports after 100 doses sold compared to 100 ADR reports for Test drug after 1000 doses sold, it appears that Reference drug is safer based on these apparent ADR statistics. Test drug has an ADR reporting rate of one in ten doses when exposure is taken into account, while Drug B has an ADR reporting rate of one in two doses. On this basis, Test drug is therefore relatively safer.

To make such comparisons more robust, adjustments should be made for differences in drug exposure. This can be achieved by normalizing ADR counts based on annual sales or a similar proxy measure. Assuming comparable spontaneous reporting rates for the drugs in question, this method allows for a more accurate assessment of relative safety.

In cases where ADR counts exhibit exponential or sigmoidal growth patterns, the growth rate parameter (β) can be utilized for comparison. Applying these methodologies to specific drug categories, such as cancer drugs, can provide insightful safety evaluations and support more informed decision-making in drug selection.

## 4 Practical implications

### 4.1 Safety comparison of cancer drugs

Trends in the ADR counts of illegal drugs might be significant, but it's more interesting to compare prescription medications that have previously been prescribed using this method.

We have examined the situation with cancer treatment drugs, a rigorous examination of their safety is paramount due to the multifaceted nature of their use and the high risk associated with their side effects. This study will involve a comparative analysis of various cancer therapies, focusing on their adverse effects, interactions with other medications, and long-term health outcomes. The research technique will assess the frequency and severity of adverse drug reactions (ADRs) by gathering and analyzing data from clinical trials, patient reports, and real-world evidence. Special attention will be given to drug interactions, particularly in polypharmacy scenarios common among cancer patients, and the impact of these interactions on overall safety. Additionally, the study will investigate long-term health consequences associated with cancer treatments, including potential secondary cancers and chronic conditions. By employing statistical models and safety assessment frameworks, this research aims to identify and mitigate risks, thereby informing more effective and personalized treatment strategies while enhancing patient safety and well-being.

Therefore, our goal is to compare its ADR count to that of others. In every instance, an exponential model was fitted well. Table 2 displays the outcome, a novel metric introduced in this work.

**Table 2:** Safety Ranking of the cancer drugs (Cumulative ADR Observed vs. Fitted).

Drug Name	Equation	SE	AIC	BIC	RMSE	R^2^	Rank (β)
Tamoxifen	y = 1198.5xe^0.0972x^	0.0022	469.2903	472.3430	936.8332	0.95	0.0972
Avastin	y = 2215.9xe^0.1211x^	0.0029	565.2778	568.3305	3843.2961	0.92	0.1211
Bleomycin	y = 114.55xe^0.139x^	0.0022	469.2903	472.3430	936.8332	0.979	0.139
Paclitaxel	y = 1526.4xe^0.1673x^	0.0016	436.5607	439.2952	1733.5786	0.998	0.1673
Vincristine	y = 210.66xe^0.1706x^	0.0041	507.3868	510.4395	1640.4856	0.98	0.1706
Methotrexate	y = 438.1xe^0.1853x^	0.0074	622.3788	625.4315	8900.0016	0.971	0.1853
Cisplatin	y = 331.75xe^0.1859x^	0.0041	563.0777	566.1304	3720.9365	0.973	0.1859
Doxorubicin	y = 922.11xe^0.208x^	0.0076	437.0841	439.5218	5777.2418	0.91	0.208
Imatinib	y = 661.54xe^0.2427x^	0.0092	375.6955	377.8776	4663.9854	0.85	0.2427
Docetaxel	y = 174.39xe^0.267x^	0.0063	514.7206	517.4552	6671.0245	0.888	0.267
Rituxima	y = 243.38xe^0.2771x^	0.0142	381.3210	383.7621	1893.9258	0.91	0.2771
Trastuzumab	y = 64.147xe^0.3025x^	0.0144	336.4257	338.8635	771.6375	0.87	0.3025
Revlimid	y = 67.908xe^0.5431x^	0.0106	372.1650	374.0540	16,131.9053	0.72	0.5431
Lenalidomide	y = 67.848xe^0.5432x^	0.0236	343.4810	345.3621	7582.3512	0.72	0.5432
Pembrolizumab	y = 1.1524xe^0.8277x^	0.0344	221.370	222.7860	1402.0260	0.73	0.8277

All ADR data for cancer drugs were sourced from FDA Adverse Event Reporting System (FAERS) and annual ADR counts were extracted continuously from drug approval year until 2023.

In all cases, the exponential growth model demonstrated a good fit for the ADR data, with satisfactory residual plots validating the model's performance. Within this framework, the exponential growth rate parameter (β) plays a crucial role in determining the shape of the ADR growth curve, reflecting the rate at which ADR counts increase over time. Specifically, a lower value of (β) indicates a slower rate of ADR growth, suggesting a safer drug. Importantly, this parameter is independent of the drug's usage extent, meaning that the growth rate (β) is solely indicative of the drug's relative safety profile, regardless of how widely the drug is used.

If the usage of a drug is doubled and all counts of adverse drug reactions (ADRs) also double, the growth rate parameter (β) remains unchanged. This is because r specifically measures the relative rate of growth of ADR reports and is independent of the scale of the data. The doubling affects only the parameter α, which represents the initial level of ADR counts.

In the case of evaluating the safety profile of sevoflurane, despite its high efficacy and low growth rate (β), it appears less favorable compared to three other drugs, which demonstrate a superior safety profile based on the same growth rate measure. This unexpected finding highlights the need for further investigation by experts in the field. Understanding the nuances behind these results could provide deeper insights into the safety dynamics and help refine safety evaluations of cancer drugs and other drugs.

### 4.2 Limitations

Concurrent medication uses and drug–drug interactions (DDIs) might increase or decrease the safety profile of a discontinued medicine, affecting ADR patterns. Polypharmacy, especially in older persons and chronically ill patients, can increase the risk of pharmacokinetic or pharmacodynamic interactions that cause ADRs unrelated to the discontinued drug. Research indicates that DDIs considerably contribute to preventable ADRs in real-world situations and can confound post-marketing safety evaluations.^23^, 24.

Residual use, stored drugs, continuous prescriptions, and delayed adverse events might create legitimate ADRs years after discontinuation. Studies have shown that medications with lengthy half-lives or sluggish physiological recovery processes might cause delayed or extended ADR onset.^25^ Increased reporting after regulatory notifications may temporarily increase ADR counts.^26^

Given these considerations, this study's post-withdrawal ADR patterns should be taken with caution, as some late reports may be due to co-medication effects, DDIs, or real delayed adverse consequences.

This study presents preliminary, not causative, insights. The observed trends in ADR increase and reporting are simply descriptive and meant to generate hypotheses. Because spontaneous reporting data include inherent biases and lack controlled exposure details, the findings cannot be used to establish cause-and-effect relationships and require more investigation.

### 4.3 Further scope of research

Utilize machine learning and artificial intelligence to identify patterns and predict ADRs, potentially improving early detection of safety signals. Conduct longitudinal studies to track ADRs over extended periods after a drug's withdrawal, providing insights into long-term safety profiles. Develop a common decision-making probability model underlying safety of different drugs and some simulations results to improve patient's quality of life and well-being.

Future research should use time-to-event analysis to examine the time between medication administration and adverse drug reaction (ADR) reporting. This is essential because annual ADR totals couldn't establish these occurrences' timing. Validation must use exposure-adjusted adverse drug reaction (ADR) rates. This strategy improves safety comparison interpretation beyond basic reporting trends.

## Generative AI statement

The author(s) declare that no Gen AI was used in the creation of this manuscript.

## CRediT authorship contribution statement

**Samadhan Ghubade:** Writing – review & editing, Validation, Software, Resources, Project administration, Methodology, Investigation, Formal analysis, Data curation, Conceptualization. **Sharvari Shukla:** Writing – review & editing, Supervision, Methodology, Investigation.

## Ethical consideration

All data were derived from public, de-identified pharmacovigilance databases.

Therefore, no ethical approval or informed consent was required.

## Funding

The author(s) declare that no financial support was received for the research and/or publication of this article.

## Declaration of competing interest

The authors declare that they have no known competing financial interests or personal relationships that could have appeared to influence the work reported in this paper.

## Data Availability

The corresponding author can provide the datasets used and analyzed in this study upon reasonable request. The FDA's Adverse Event Reporting System (FAERS) and Public Dashboard (https://www.fda.gov/drugs/fdas-adverse-event-reporting-system-faers/fda-adverse-event-reporting-system-faers-public-dashboard), as well as VigiAccess (www.vigiaccess.org), the WHO's global database of reported possible drug side effects, were the sources of the data.
